# Supplementary figures and images for: Does Catheter Insertion Site Matter? Contamination of Peripheral Intravenous Catheters during Dental Scaling in Dogs
Source: Vet Sci. 2024 Sep 3;11(9):407. doi: 10.3390/vetsci11090407 (PMC11435992; doi:10.3390/vetsci11090407)

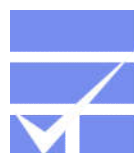

# CONSORT

TRANSPARENT REPORTING of TRIALS

## CONSORT 2010 Flow Diagram

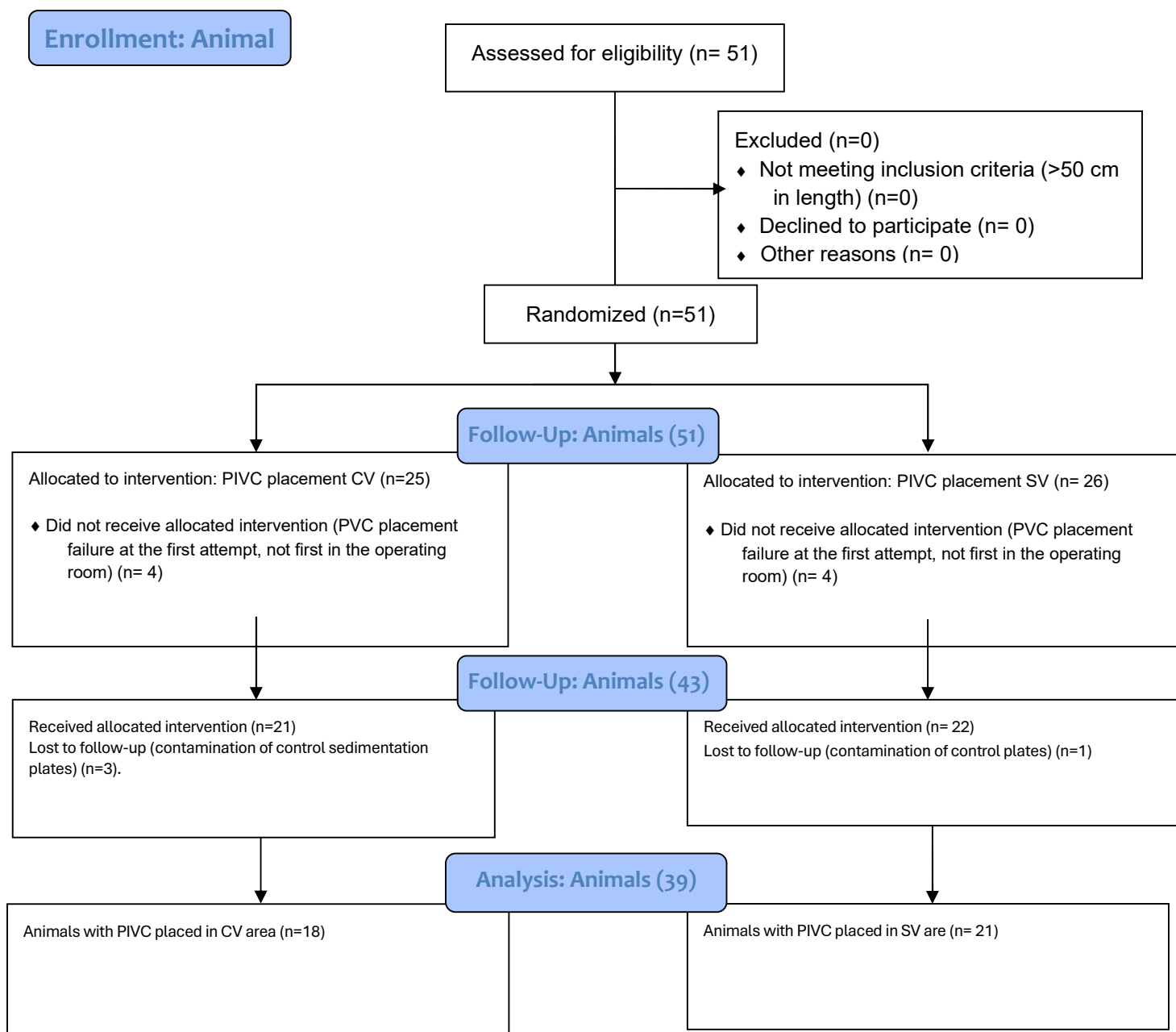

Supplement: Supplementary file 1 [file vetsci-11-00407-s001.zip › vetsci-3061279-supplementary.pdf]
